# Anomalous left coronary artery from the pulmonary artery: Outcomes and management of mitral valve

**DOI:** 10.3389/fcvm.2022.953420

**Published:** 2022-10-06

**Authors:** Juemin Yu, Qiushi Ren, Xiaobing Liu, Tianyu Chen, Rong Liufu, Shusheng Wen, Jimei Chen, Jianzheng Cen, Jian Zhuang

**Affiliations:** ^1^Department of Cardiovascular Surgery, Guangdong Cardiovascular Institute, Guangdong Provincial People’s Hospital, Guangdong Academy of Medical Sciences, Guangzhou, China; ^2^School of Medicine, South China University of Technology, Guangzhou, China

**Keywords:** congenital heart disease, anomalous left coronary artery from the pulmonary artery (ALCAPA), mitral regurgitation, concomitant mitral valve repair, outcome

## Abstract

**Objective:**

Use of concomitant mitral valve repair remains controversial in the anomalous left coronary artery from the pulmonary artery (ALCAPA) with mitral regurgitation (MR). This study aimed to evaluate postoperative mitral valve function and explore the indication for concomitant mitral valve repair.

**Materials and methods:**

The medical records of 111 patients with ALCAPA and MR who underwent ALCAPA surgery between April 2006 and November 2020 were reviewed. The patients were categorized into three groups for comparison, namely, group I consisted of 38 patients with trivial or mild MR who underwent ALCAPA repair only; group II consisted of 37 patients with moderate or severe MR who similarly had only surgery of the ALCAPA performed; and group III consisted of 36 patients who had concomitant mitral valve repair for moderate or severe MR.

**Result:**

Overall mortality was 7.2% (8 of 111). The mortality of group II (16.2%, 6 of 37) was higher than those of groups I (5.3%, 2 of 38) and III (0%, 0 of 36) (*p* = 0.027). All three patients who underwent mitral valve reintervention were in group II. At the last follow-up, none of the patients had more than moderate MR in group I. The percentage of patients with improved MR grade was 79.4% (27 of 34) in group III and 51.4% (19 of 37) in group II (*p* = 0.001). The multivariate logistic regression revealed that concomitant mitral valve repair (adjusted odds ratio = 4.492, 95% CI: 1.909–12.794; *p* < 0.001) was the major factor influencing MR grade improvement.

**Conclusion:**

The long-term outcomes after ALCAPA repair were favorable. For mild MR, ALCAPA repair only can be performed. For moderate and severe MR, we suggest concomitant mitral valve repair.

## Introduction

The anomalous left coronary artery from the pulmonary artery (ALCAPA) is a rare congenital anomaly of coronary anatomy, with an incidence of approximately 1 in 300,000 births (1, 2). In the first few weeks of life due to the closure of the ductus arteriosus, pressure in the pulmonary arteries decreases, pulmonary vascular resistance decreases, and LCA perfusion decreases. These changes are associated with a range of adverse outcomes, such as left ventricular dysfunction, left ventricular dilatation, and mitral regurgitation (MR).

Currently, surgical treatment of ALCAPA has positive outcomes. However, whenever MR is present, the decision to intervene has always been controversial. Brown et al. (3) argued that concurrent mitral valve intervention is unnecessary for patients with ALCAPA. However, Biçer et al. (4) noted that despite the low rate of mitral valve reintervention, more than moderate preoperative MR required attention. Similarly, Weixler et al. (5) believed that patients with more than moderate MR had a higher risk of reintervention after surgery. This study aimed to evaluate the long-term outcome of mitral valve repair and explore the indications for mitral valve intervention by reviewing cases at our center.

## Materials and methods

From April 2006 to November 2020, 128 patients underwent ALCAPA repair at Guangdong Provincial People’s Hospital. After reviewing the medical records of all potential cases, 111 cases were obtained for this study. Two patients who underwent mitral valve repair due to MR prior to ALCAPA repair were excluded. In addition, two other patients without preoperative echocardiographic records were excluded. Seven patients had normal mitral valve function before the operation, two patients had undergone the Takeuchi procedure, three patients had undergone left coronary ligation with coronary bypass surgery, and one patient had undergone left coronary ligation without coronary bypass surgery. The following data were retrieved from the clinical records: demographic variables, preoperative and postoperative transthoracic echocardiographic findings, surgical findings, and further interventions required after the initial operation. This study was approved by the ethics committee of Guangdong Provincial People’s Hospital on 12 September 2019 [Approval ID No.: GDREC2019338H(R2)]. The approval included a waiver of informed consent.

Based on the severity of MR and the surgical procedures performed, the patients were categorized into three groups for comparison. Group I consisted of 38 patients with trivial or mild MR who underwent ALCAPA repair only. Group II consisted of 37 patients with moderate or severe MR who similarly had only surgery of the ALCAPA performed. Group III consisted of 36 patients who had moderate or severe MR and underwent concomitant mitral valve repair in addition to ALCAPA repair.

Echocardiogram Z-scores of MR, left ventricular ejection fraction (LVEF), and left ventricular end-diastolic diameter (LVEDD) were obtained (6). The degree of MR was graded as none, trivial, mild, moderate, or severe (7). Echocardiography was performed before operation, 1 week before discharge, and 3 and 6 months after the operation to evaluate coronary and pulmonary artery stenosis, ventricular function, and MR. Subsequently, all patients underwent regular echocardiography examinations annually. The severity of MR at the last follow-up was used in the outcome analysis. Improvement in the degree of MR was defined as a reduction in the degree of MR at the most recent follow-up compared with the preoperative MR. Early postoperative death was defined as predischarge hospital death, and long-term death was defined as postdischarge death.

All patients underwent surgical correction by median sternotomy. The aorta was cross-clamped, and the right and left pulmonary arteries, superior vena cava, and inferior vena cava were snared. Cardioplegia was induced in the aortic root and main pulmonary artery. The right atrium was incised, the pulmonary artery was transected, the orifice of the LCA was determined, and the LCA position was observed. The LCA button was clipped from the pulmonary artery, and an appropriate size was cut in an appropriate aortic location for coronary reimplantation. For patients with moderate or severe MR, the decision to perform concomitant mitral valve intervention was made according to surgeons’ preferences and MR degree. A total of 36 (32.4%) patients underwent concomitant mitral valve intervention. Patients in group III underwent different techniques for mitral valve repair (Table 1).

**TABLE 1:** MV pathology and operative techniques of MV repair in all 36 patients of group III.

MV pathology	No. of patients	MV repair technique
Ring dilatation	22	Annuloplasty with mattress
Prolapse of anterior leaflet, ring dilatation	5	Annuloplasty with mattress
Prolapse of anterior leaflet	1	Annuloplasty with mattress
Prolapse of A2 and A3	1	Mitral valvuloplasty with Gore-Tex suture as an artificial chordae tendineae
Ring dilatation, Ischemia of papillary muscle	1	Annuloplasty with mattress
Ischemia of papillary muscle	1	Annuloplasty with mattress
prolapse of mitral valve	1	Annuloplasty with mattress
Partial adhesion of subvalvular chordae tendon	2	Mechanical Valve Implantation
N/A	2	N/A

MV, mitral valve; N/A, not applicable.

Continuous variables are expressed as mean and standard deviation for normally distributed data or median and range for non-normally distributed data. Data were assessed for normality of distribution using the Shapiro–Wilk test. Differences in normally distributed variables among the three groups were determined using a one-way analysis of variance with *post-hoc* comparisons using the Bonferroni test. Furthermore, analysis of non-normally distributed data was performed using the Kruskal–Wallis test with *post-hoc* comparisons using Dunn’s multiple comparison test. The classification variables are represented by appropriate frequencies or percentages, and the intergroup differences of variables were analyzed using Fisher’s exact test. Confounders were controlled using multivariate logistic regression. A *p*-value of < 0.05 was considered statistically significant. All reported *p*-values were bilateral. All data were analyzed using SPSS version 26.0 (Chicago, Illinois SPSS).

## Results

### Baseline characteristics

The preoperative characteristics of all patients are summarized in Table 2. The median age at surgery was 9 months (range: 1 month to 44 years), and the median follow-up period was 5.5 years (range: 0.5–15.03 years). Patients in group I were older and heavier than those in group II (*p* = 0.007, *p* = 0.009). Group I had the lowest LVEDD Z-score among the three groups (*p* = 0.003). Fewer patients in group I required preoperative inotropic support than in group III (*p* = 0.008). Age at the time of surgery, weight at the time of surgery, preoperative LVEF, and preoperative LVEDD were comparable between groups II and III.

**TABLE 2:** The preoperative characteristics of all patients.

Characteristic	All (*n* = 111)	Group I (*n* = 38)	Group II (*n* = 37)	Group III (*n* = 36)	*p*-value
Female gender, n	69 (62.2)	23 (60.5)	23 (62.2)	23 (63.9)	0.969
Age at surgery, years	0.75 (0.08, 49)	3.5 (0.08, 42)^a^	0.58 (0.17, 37)^b^	0.67 (0.08, 44)^a,b^	0.007
Weight at surgery, kg	7.5 (3.5, 67)	12.75 (3.5, 67)^a^	6.5 (3.8, 48.5)^b^	6.75 (4.2, 50.5)^a,b^	0.009
Positive inotropic drugs, n	54 (48.6)	11 (28.9)^a^	20 (54.1)^a,b^	23 (63.9)^b^	0.08
Mechanical ventilation, n	7 (6.3)	3 (7.9)	3 (8.1)	1 (2.8)	0.696
LVEDD *Z*-score	3.93 (–3.97, 7.89)	1.68 (–3.97, 7.32)^a^	4.67 (–1.09, 7.64)^b^	4.29 (0.25, 7.89)^b^	0.003
LVEF (%)	53 (16, 86)	62 (16, 78)	44 (20, 86)	58.5 (21, 78)	0.118
**MR**					< 0.001
Trivial	11 (9.9)	11 (28.9)	0^b^	0^b^	
Mild	27 (24.3)	27 (71.1)			
Moderate	45 (40.5)	0^a^	27 (73.0)^b^	18 (450)^c^	
Server	28 (25.2)	0^a^	10 (27)^b^	18 (50)^c^	

Values are presented as median (range), n (%). Each superscript letter indicates a subset of group categories whose column proportions do not differ significantly from each other at the 0.05 level. LVEDD, left ventricular end-diastolic dimension; LVEF, left ventricular ejection fraction; MR, mitral regurgitation.

### Surgical outcome

The surgical outcomes of all patients are summarized in Table 3. No differences in cardiopulmonary bypass time and aortic occlusion time were found among the three groups (*p* = 0.208, *p* = 0.130). The postoperative mechanical ventilation time of group I was less than those of groups II and III (*p* = 0.032). However, no significant difference existed between group II and group III. No difference in ICU time existed among the three groups (*p* = 0.096). Postoperative hospital stay length was shorter in group I than those in other groups (*p* = 0.009). Six patients, including one patient in group I, one patient in group III, and four patients in group II, were assisted with extracorporeal membrane oxygenation (ECMO) after surgery (*p* = 0.319).

**TABLE 3:** Surgical outcomes.

Variable	Group I	Group II	Group III	*p*-value
Aortic cross-clamping time (min)	78 (38, 250)	75 (25, 149)	76 (53, 135)	0.130
Cardiopulmonary bypass time (min)	144.5 (66, 517)	136 (58, 532)	159.5 (76.354)	0.208
Mechanical ventilation time (h)	16 (3, 528)^a^	57.25 (4, 476)^b^	50.25 (5, 696)^b^	0.032
Intensive care unit stay (d)	3 (1, 38)	4 (1, 47)	4 (1, 41)	0.096
Postoperative hospital stays (d)	8 (2, 45)^a^	14 (5, 55)^b^	15 (4, 65)^b^	0.009
Mortality	2 (5.6)^a,b^	6 (16.2)^a^	0^b^	0.027
Reoperation	0^a^	3 (9.4)^b^	0^a^	0.031

Values are presented as median (range), n (%). Each superscript letter indicates a subset of group categories whose column proportions do not differ significantly from each other at the 0.05 level.

The median follow-up period was 5.49 years (range: 0.5–15.03 years). A total of six early deaths and two late deaths were recorded, with a total mortality rate of 7.2%. Two patients each in groups I and III were lost to follow-up during the follow-up period. One patient had mild MR preoperatively in group I and died postoperatively on the 7th day due to severe low cardiac output syndrome. One patient died after 158 days. This outcome was obtained by telephone follow-up; therefore, the specific cause of death was not ascertained. In addition, none of the patients had mitral valve reintervention. In group II, five patients died early, three patients died postoperatively within 5 days due to low cardiac output syndrome, and two patients died postoperatively on the 7th and 9th days due to sudden cardiac arrest secondary to ventricular fibrillation. Among the patients who died early, three patients had severe MR preoperatively, two patients had moderate MR preoperatively, and one patient had mild MR preoperatively. One patient died after 359 days during follow-up; this outcome was obtained by telephone follow-up. Three patients underwent mitral valve surgery due to severe MR at 2, 3, and 4 years after the initial surgery. From the intraoperative, postoperative, and follow-up echocardiography, none of the patients had coronary artery stenosis and pulmonary artery stenosis. Group III had no deaths and reinterventions. Group II had a higher mortality rate than group III (6/37 vs. 0/34; *p* = 0.027), and group II had a higher rate of long-term mitral valve reintervention than group III (3/31 vs. 0/34; *p* = 0.031).

### Left ventricular function

In group I, the median LVEF was 62% (range = 16–78%) before surgery, 56% (range = 12–73%) before discharge, and 69% (range = 32–82%) at the last follow-up. LVEF at the most recent follow-up was better than those recorded preoperatively and before discharge (*p* < 0.001; *p* < 0.001). Compared with preoperative LVEF, postoperative LVEF decreased (*p* = 0.003). The preoperative median LVEDD Z-score, 1.69 (range = –3.97 to 7.32), was poorer than the postoperative median LVEDD Z-score, 0.24 (range = –3.46 to 7.56) (*p* < 0.001).

In group II, the median LVEF was 44.5% (range = 20–86%) before surgery, 56% (range = 15–80%) before discharge, and 69% (range = 32–82%) at the last follow-up. LVEF at the most recent follow-up was better than those recorded preoperatively and before discharge (*p* < 0.001; *p* < 0.001). Compared with the preoperative LVEF, the median LVEF at discharge was not different (*p* = 0.068). The preoperative median LVEDD Z-score, 4.67 (range = –1.09 to 7.64), was poorer than the postoperative median LVEDD Z-score, 1.32 (range = –3.67 to 5.61) (*p* < 0.001).

In group III, the median LVEF was 58.5% (range = 21–78%) before surgery, 54.5% (range = 11–79%) before discharge, and 68% (range = 19–85%) at the last follow-up. LVEF at the recent follow-up was better than those recorded preoperatively and before discharge (*p* < 0.001; *p* = 0.004). Compared with preoperative LVEF, postoperative LVEF decreased (*p* = 0.042). The preoperative median LVEDD Z-score, 4.297 (range = 0.25–7.89), was poorer than the postoperative median LVEDD Z-score, 1.57 (range = –4.26 to 4.61) (*p* < 0.001).

No significant differences in LVEF were observed among the three groups preoperatively and at the last follow-up (*p* = 0.085; *p* = 0.774; *p* = 0.638). Patients in group I had lower LVEDD Z-scores than those in other two groups (*p* = 0.003), and no significant differences in LVEDD Z-scores were observed among the three groups after surgery.

### Mitral valve function

**FIGURE 1:**
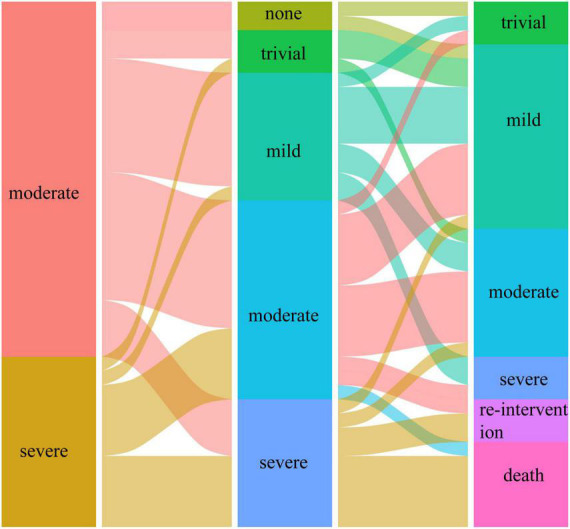
The changes in mitral regurgitation among preoperative, postoperative, and last follow-up periods in group II.

**FIGURE 2:**
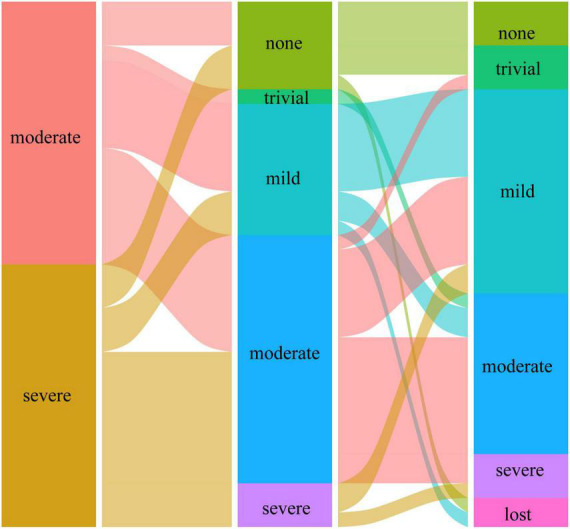
The changes in mitral regurgitation among preoperative, postoperative, and last follow-up periods in group III.

Preoperatively, the MR grade was trivial in 11 (28.9%) patients and mild in 27 (71.1%) patients in group I. At the last follow-up, none of the patients in group I had more than moderate MR and underwent reintervention for MR. Figures 1, 2 illustrate the changes in MR among the preoperative, postoperative, and last follow-up periods for groups II and III. Preoperatively, 27 and 18 patients had moderate MR in groups II and III, respectively. A total of 10 and 18 patients had severe MR in groups II and III, respectively. At the last follow-up, three patients had trivial MR, 13 patients had mild MR, nine patients had moderate MR, and three patients had severe MR in group II. Three patients had no MR, three patients had mild MR, 14 patients had moderate MR, and three patients had severe MR in group III. The percentage of patients with improved MR grades was 79.4% (27 of 34 patients) in group III and 51.4% (19 of 37 patients) in group II (*p* = 0.001). Multivariate logistic regression findings for independent risk factors for the MR grade improvement are provided in Table 4. From the multivariate logistic regression analysis, concomitant mitral valve repair (adjusted odds ratio = 4.492, 95% CI: 1.909–12.794; *p* < 0.001) was the major factor influencing MR grade improvement.

**TABLE 4:** Multivariate analysis for the degree of mitral regurgitation improved after the operation.

Variable	Odds ratio	95% CI	*p*-value
Age	0.943	0.844–1.054	0.300
Weight	1.045	0.974–1.121	0.217
Concomitant MV repair	5.889	2.132–16.263	0.001
LVEF	1.008	0.973–1.044	0.656
LVEDD *Z*-score	1.131	0.853–1.499	0.394
MR*			
Mild	1.042	0.227–4.779	0.958
Moderate	2.049	0.421–9.986	0.374
Server	3.013	0.523–17.366	0.217

MV, mitral valve; LVEDD, left ventricular end-diastolic dimension; LVEF, left ventricular ejection fraction; MR, mitral regurgitation. *Odds ratios for patients with preoperative MR of other grades compared with preoperative MR of trivial.

## Discussion

The main finding of this study is that for patients with ALCAPA and moderate or severe MR, concomitant mitral valve repair in ALCAPA repair is feasible. Our data suggest that concomitant mitral valve repair should be performed in patients with ALCAPA and moderate and severe MR. The reasons are as follows:

1. For mild MR, even without mitral valve intervention, no MR function deterioration or MR reintervention was needed.
2. Mitral function was better in patients who underwent concomitant mitral valve repair than in those who underwent ALCAPA repair only (79.4% vs. 51.4%; *p* = 0.008).
3. Patients who underwent concomitant mitral valve repair had higher survival rate and reintervention exclusion rate (83.8% vs. 100%, *p* = 0.027; 90.6% vs. 100%, *p* = 0.031; respectively).

Our data suggest that concomitant mitral valve repair for ALCAPA with moderate or severe MR had no poor early outcomes than ALCAPA repair only. However, some clinicians (3, 8) believe that concomitant mitral valve repair will prolong the aortic occlusion time, increasing postoperative mortality and postoperative complications. In our cohort, the aortic occlusion time and cardiopulmonary bypass time of patients who underwent concomitant mitral valve intervention were similar to those of other patients who underwent ALCAPA repair only. No significant differences in postoperative mechanical ventilation time and postoperative ICU time existed between groups II and III. In addition, no mortality was recorded in group III. According to Triglia et al. (9), Alexi et al. (10), and Isomatsu et al. (11), concomitant mitral valve repair does not result in adverse consequences after the operation. Other studies reported that mitral valve repair can improve the early postoperative cardiac output and can be conducive to early recovery of cardiac function (12, 13). Concomitant mitral valve repair for moderate or severe MR does not result in poor outcomes in the early postoperative period and has similar perioperative recovery.

Our data suggest that patients with ALCAPA and moderate or severe MR who did not undergo concomitant mitral valve intervention had higher mortality and reintervention rates than those who underwent concomitant mitral valve intervention. In our study, the total mortality was 7.2%, of which 5.4% occurred prior to discharge from the hospital. This finding is similar to findings from studies reported in recent years (14–17). The long-term reintervention rate was 3%. One case of moderate MR and two cases of severe MR were recorded among patients who underwent reintervention. Before reintervention, these patients had severe MR, suggesting that some patients with moderate MR may progress even after ALCAPA repair only. In our cohort, postoperative death and mitral valve reintervention were both present in group II, and no cases of death and reintervention were reported in group III. Weixler et al. (5) reported an early mortality rate of 6.9% and a mitral valve reintervention rate of 10.25% in patients who underwent ALCAPA repair only. Furthermore, no death and mitral valve reintervention were reported in the surgical group during the same period. Zhang et al. (18) indicated that concurrent mitral valve repair could result in better postoperative recovery and higher survival rate.

The MR improvement in concomitant mitral valve repair was significantly higher than in ALCAPA repair only. The number of patients with improved MR in group III was more than that in group II. Moreover, more patients with severe preoperative MR in group III underwent concomitant mitral valve repair. These data suggest an association between concomitant mitral valve repair and mitral valve improvement. Furthermore, our multivariate analysis of MR grade improvement revealed that concomitant mitral valve repair was a major factor influencing the long-term improvement of MR degree, which further confirmed our inference. We speculate that for moderate and severe MR, the extent of damage to the mitral valve is high that the mere correction of anomalous coronary arteries and recovery of the double coronary flow are insufficient for the functional recovery of the mitral valve, requiring a concomitant mitral valve repair. In addition, Weixler et al. (5) reported similar improvements in mitral valve function, with 89% in the concomitant mitral valve repair group and 41% in the no mitral valve repair group.

In this study, no significant differences in the recovery of ventricular function existed among the three groups. After ALCAPA repair, the left ventricular function of most patients returned to normal regardless of concomitant mitral valve intervention, which is similar to that reported in most literature (19–21). In addition, we found that the left ventricular diameter of most patients improved significantly before discharge, and the LVEF did not improve immediately. This may mean that ventricular morphology recovery is faster than function recovery after ALCAPA repair. However, this study was retrospective, and this conclusion needs verification by further studies. Cochrane et al. (22) and Imamura et al. (23) reported similar results on left ventricular recovery. In group III, the early postoperative LVEF was lower than the preoperative LVEF. We considered this because MR was reduced, and LV afterload, ejection resistance, and LV myocardial contractility were restored after concomitant mitral valve repair, resulting in a corresponding decrease in ejection fraction.

This study had some limitations which should be noted. The critical limitation was that this study was retrospective and participant selection was non-randomized. In this study, we could not preclude results being influenced by differences between the surgical period and surgeon, which may limit meaningful comparison of outcomes between concomitant mitral valve repair and no repair. In addition, some patients failed to complete the examinations on time when they were followed up. Therefore, some data were missing.

## Conclusion

Surgical treatment of ALCAPA has good efficacy, with acceptable mortality and an expected reintervention rate. Postoperative cardiac function mostly recovers, but ventricular diameter recovers faster than ejection fraction. For the management of the mitral valve, we recommend concomitant mitral valve repair for moderate or severe MR and ALCAPA repair only for mild and trivial MR.

## Data availability statement

The original contributions presented in this study are included in the article/supplementary material, further inquiries can be directed to the corresponding author.

## Ethics statement

The studies involving human participants were reviewed and approved by the Ethics Committee of Guangdong Provincial People’s Hospital. Written informed consent to participate in this study was provided by the participants’ legal guardian/next of kin. Written informed consent was obtained from the individual(s), and minor(s)’ legal guardian/next of kin, for the publication of any potentially identifiable images or data included in this article.

## Author contributions

JY, XL, and QR wrote the manuscript and performed the statistical analysis. TC and RL performed the data inspection and validation. JMC and SW provided funding support and supervision. JZC and JZ revised the manuscript. All authors contributed to the article and approved the submitted version.
